# Health Recommender Systems: Systematic Review

**DOI:** 10.2196/18035

**Published:** 2021-06-29

**Authors:** Robin De Croon, Leen Van Houdt, Nyi Nyi Htun, Gregor Štiglic, Vero Vanden Abeele, Katrien Verbert

**Affiliations:** 1 Department of Computer Science KU Leuven Leuven Belgium; 2 Faculty of Health Sciences University of Maribor Maribor Slovenia

**Keywords:** health recommender systems, recommender, recommendation system, health, health care, patient, layperson, systematic review, eHealth, evaluation, recommender technique, user interface, guidelines, mobile phone

## Abstract

**Background:**

Health recommender systems (HRSs) offer the potential to motivate and engage users to change their behavior by sharing better choices and actionable knowledge based on observed user behavior.

**Objective:**

We aim to review HRSs targeting nonmedical professionals (laypersons) to better understand the current state of the art and identify both the main trends and the gaps with respect to current implementations.

**Methods:**

We conducted a systematic literature review according to the PRISMA (Preferred Reporting Items for Systematic Reviews and Meta-Analyses) guidelines and synthesized the results. A total of 73 published studies that reported both an implementation and evaluation of an HRS targeted to laypersons were included and analyzed in this review.

**Results:**

Recommended items were classified into four major categories: lifestyle, nutrition, general health care information, and specific health conditions. The majority of HRSs use hybrid recommendation algorithms. Evaluations of HRSs vary greatly; half of the studies only evaluated the algorithm with various metrics, whereas others performed full-scale randomized controlled trials or conducted in-the-wild studies to evaluate the impact of HRSs, thereby showing that the field is slowly maturing. On the basis of our review, we derived five reporting guidelines that can serve as a reference frame for future HRS studies. HRS studies should clarify who the target user is and to whom the recommendations apply, what is recommended and how the recommendations are presented to the user, where the data set can be found, what algorithms were used to calculate the recommendations, and what evaluation protocol was used.

**Conclusions:**

There is significant opportunity for an HRS to inform and guide health actions. Through this review, we promote the discussion of ways to augment HRS research by recommending a reference frame with five design guidelines.

## Introduction

### Research Goals

Current health challenges are often related to our modern way of living. High blood pressure, high glucose levels, and physical inactivity are all linked to a modern lifestyle characterized by sedentary living, chronic stress, or a high intake of energy-dense foods and recreational drugs [1]. Moreover, people usually make poor decisions related to their health for distinct reasons, for example, busy lifestyles, abundant options, and a lack of knowledge [2]. Practically, all modern lifestyle health risks are directly affected by people’s health decisions [3], such as an unhealthy diet or physical inactivity, which can contribute up to three-fourth of all health care costs in the United States [4]. Most risks can be minimized, prevented, or sometimes even reversed with small lifestyle changes. Eating healthily, increasing daily activities, and knowing where to find validated health information could lead to improved health status [5].

Health recommender systems (HRSs) offer the potential to motivate and engage users to change their behavior [6] and provide people with better choices and actionable knowledge based on observed behavior [7-9]. The overall objective of the HRS is to empower people to monitor and improve their health through technology-assisted, personalized recommendations. As one approach of modern health care is to involve patients in the cocreation of their own health, rather than just leaving it in the hands of medical experts [10], we limit the scope of this paper to HRSs that focus on laypersons, for example, nonhealth care professionals. These HRSs are different from clinical decision support systems that provide recommendations for health care professionals. However, laypersons also need to understand the rationale of recommendations, as echoed by many researchers and practitioners [11]. This paper also studies the role of a graphical user interface. To guide this study, we define our research questions (RQs) as follows:

RQ1: What are the main applications of the recent HRS, and what do these HRSs recommend?

RQ2: Which recommender techniques are being used across different HRSs?

RQ3: How are the HRSs evaluated, and are end users involved in their evaluation?

RQ4: Is a graphical user interface designed, and how is it used to communicate the recommended items to the user?

### Recommender Systems and Techniques

Recommender techniques are traditionally divided into different categories [12,13] and are discussed in several state-of-the-art surveys [14]. *Collaborative filtering* is the most used and mature technique that compares the actions of multiple users to generate personalized suggestions. An example of this technique can typically be found on e-commerce sites, such as “Customers who bought this item also bought...” *Content-based filtering* is another technique that recommends items that are similar to other items preferred by the specific user. They rely on the characteristics of the objects themselves and are likely to be highly relevant to a user’s interests. This makes content-based filtering especially valuable for application domains with large libraries of a single type of content, such as MedlinePlus’ curated consumer health information [15]. *Knowledge-based filtering* is another technique that incorporates knowledge by logic inferences. This type of filtering uses explicit knowledge about an item, user preferences, and other recommendation criteria. However, knowledge acquisition can also be dynamic and relies on user feedback. For example, a camera recommender system might inquire users about their preferences, fixed or changeable lenses, and budget and then suggest a relevant camera. *Hybrid recommender systems* combine multiple filtering techniques to increase the accuracy of recommendation systems. For example, the *companies you may want to follow* feature in LinkedIn uses both content and collaborative filtering information [16]: collaborative filtering information is included to determine whether a company is similar to the ones a user already followed, whereas content information ensures whether the industry or location matches the interests of the user. Finally, recommender techniques are often augmented with additional methods to incorporate contextual information in the recommendation process [17], including recommendations via contextual prefiltering, contextual postfiltering, and contextual modeling [18].

### HRSs for Laypersons

Ricci et al [12] define recommender systems as:

Recommender Systems (RSs) are software tools and techniques providing suggestions for items to be of use to a user [13,19,20]. The suggestions relate to various decision-making processes, such as what items to buy, what music to listen to, or what online news to read.

In this paper, we analyze how recommender systems have been used in health applications, with a focus on laypersons. Wiesner and Pfeifer [21] broadly define an HRS as:

a specialization of an RS [recommender system] as defined by Ricci et al [12]. In the context of an HRS, a recommendable item of interest is a piece of nonconfidential, scientifically proven or at least generally accepted medical information.

Researchers have sought to consolidate the vast body of literature on HRSs by publishing several surveys, literature reviews, and state-of-the-art overviews. Table 1 provides an overview of existing summative studies on HRSs that identify existing research and shows the number of studies included, the method used to analyze the studies, the scope of the paper, and their contribution.

**Table 1:** An overview of the existing health recommender system overview papers.

Review	Papers, n	Method	Scope	Contribution
Sezgin and Özkan (2013) [22]	8	Systematic review	Provides an overview of the literature in 2013	Identifying challenges (eg, cyber-attacks, difficult integration, and data mining can cause ethical issues) and opportunities (eg, integration with personal health data, gathering user preferences, and increased consistency)
Calero Valdez et al (2016) [23]	17	Survey	Stresses the importance of the interface and HCI^a^ of an HRS^b^	Providing a framework to incorporate domain understanding, evaluation, and specific methodology into the development process
Kamran and Javed (2015) [24]	7	Systematic review	Provides an overview of existing recommender systems with more focus on health care systems	Proposing a hybrid HRS
Afolabi et al (2015) [25]	22	Systematic review	Research empirical results and practical implementations of HRSs	Presenting a novel proposal for the integration of a recommender system into smart home care
Ferretto et al (2017) [26]	8	Systematic review	Identifies and analyzes HRSs available in mobile apps	Identifying HRSs that do not have many mobile health care apps
Hors-Fraile et al 2018 [27]	19	Systematic review	Identifies, categorizes, and analyzes existing knowledge on the use of HRSs for patient interventions	Proposing a multidisciplinary taxonomy, including integration with electronic health records and the incorporation of health promotion theoretical factors and behavior change theories
Schäfer et al (2017) [28]	24	Survey	Discusses HRSs to find personalized, complex medical interventions or support users with preventive health care measures	Identifying challenges subdivided into patient and user challenges, recommender challenges, and evaluation challenges
Sadasivam et al (2016) [29]	15	Systematic review	Research limitations of current CTHC^c^ systems	Identifying challenges of incorporating recommender systems into CTHC. Proposing a future research agenda for CTHC systems
Wiesner and Pfeifer (2014) [21]	Not reported	Survey	Introduces HRSs and explains their usefulness to personal health record systems	Outlining an evaluation approach and discussing challenges and open issues
Cappella et al (2015) [30]	Not reported	Survey	Explores approaches to the development of a *recommendation system* for archives of public health messages	Reflecting on theory development and applications

^a^HCI: human-computer interaction.

^b^HRS: health recommender system.

^c^CTHC: computer-tailored health communication.

As can be seen in Table 1, the scope of the existing literature varies greatly. For example, Ferretto et al [26] focused solely on HRSs in mobile apps. A total of 3 review studies focused specifically on the *patient side* of the HRS: (1) Calero Valdez et al [23] analyzed the existing literature from a human-computer interaction perspective and stressed the importance of a good HRS graphical user interface; (2) Schäfer et al [28] focused on tailoring recommendations to end users based on health context, history, and goals; and (3) Hors-Fraile et al [27] focused on the individual user by analyzing how HRSs can target behavior change strategies. The most extensive study was conducted by Sadasivam et al [29]. In their study, most HRSs used knowledge-based recommender techniques, which might limit individual relevance and the ability to adapt in real time. However, they also reported that the HRS has the opportunity to use a near-infinite number of variables, which enables tailoring beyond designer-written rules based on data. The most important challenges reported were the cold start [31] where limited data are available at the start of the intervention, limited sample size, adherence, and potential unintended consequences [29]. Finally, we observed that these existing summative studies were often restrictive in their final set of papers.

Our contributions to the community are four-fold. First, we analyze a broader set of research studies to gain insights into the current state of the art. We do not limit the included studies to specific devices or patients in a clinical setting but *focus on laypersons* in general. Second, through a comprehensive analysis, we aim to identify the applications of recent HRS apps and gain insights into actionable knowledge that HRSs can provide to users (RQ1), to identify which recommender techniques have been used successfully in the domain (RQ2), how HRSs have been evaluated (RQ3), and the role of the user interface in communicating recommendations to users (RQ4). Third, based on our extensive literature review, we derive a reference frame with five reporting guidelines for future layperson HRS research. Finally, we collected and coded a unique data set of 73 papers, which is publicly available in Multimedia Appendix 1 [7-9,15,32-100] for other researchers.

## Methods

### Search Strategy

This study was conducted according to the key steps required for systematic reviews according to PRISMA (Preferred Reporting Items for Systematic Reviews and Meta-Analyses) guidelines [101]. A literature search was conducted using the ACM Digital Library (n=2023), IEEExplore (n=277), and PubMed (n=93) databases. As mentioned earlier, in this systematic review we focused solely on HRSs with a focus on laypersons. However, many types of systems, algorithms, and devices can be considered as a HRS. For example, push notifications in a mobile health app or health tips prompted by web services can also be considered as health-related recommendations. To outline the scope, we limited the search terms to include a recommender or recommendation, as reported by the authors. The search keywords were as follows, using an inclusive OR: (*recommender* OR *recommendation systems* OR *recommendation system*) AND *(health* OR *healthcare* OR *patient* OR *patients*).

In addition, a backward search was performed by examining the bibliographies of the survey and review papers discussed in the Introduction section and the reference list of included studies to identify any additional studies. A forward search was performed to search for articles that cited the work summarized in Table 1.

### Study Inclusion and Exclusion Criteria

As existing work did not include many studies (Table 1) and focused on a specific medical domain or device, such as mobile phones, this literature review used nonrestrictive inclusion criteria. Studies that met all the following criteria were included in the review: described an HRS whose primary focus was to improve health (eg, food recommenders solely based on user preferences [102] were not included); targeted laypersons (eg, activity recommendations targeted on a proxy user such as a coach [103] were not included); implemented the HRS (eg, papers describing an HRS concept are not included); reported an evaluation, either web-based or offline evaluation; peer-reviewed and published papers; published in English.

Papers were excluded when one of the following was true: the recommendations of HRSs were unclear; the full text was unavailable; or a newer version was already included.

Finally, when multiple papers described the same HRS, only the latest, relevant full paper was included.

### Classification

To address our RQs, all included studies were coded for five distinct coding categories.

#### Study Details

To contextualize new insights, the publication year and publication venue were analyzed.

#### Recommended Items

HRSs are used across different health domains. To provide details on what is recommended, all papers were coded according to their respective health domains. To not limit the scope of potential items, no predefined coding table was used. Instead, all papers were initially coded by the first author. These resulting recommendations were then clustered together in collaboration with the coauthors into four categories, as shown in Multimedia Appendix 2.

#### Recommender Techniques

This category encodes the recommender techniques that were used: collaborative filtering [104], content-based filtering [105], knowledge-based filtering [106], and their hybridizations [107]. Some studies did not specify any algorithmic details or compared multiple techniques. Finally, when an HRS used contextual information, it was coded whether they used pre- or postfiltering or contextual modeling.

#### Evaluation Approach

This category encodes which evaluation protocols were used to measure the effect of HRSs. We coded whether the HRSs were evaluated through offline evaluations (no users involved), surveys, heuristic feedback from expert users, controlled user studies, deployments *in the wild*, and randomized controlled trials (RCTs). We also coded sample size and study duration and whether ethical approval was gathered and needed.

#### Interface and Transparency

Recommender systems are often perceived as a *black box*, as the rationale for recommendations is often not explained to end users. Recent research increasingly focuses on providing transparency to the inner logic of the system [11]. We encoded whether explanations are provided and, in this case, how such transparency is supported in the user interface. Furthermore, we also classified whether the user interface was designed for a specific platform, categorized as *mobile*, *web*, or other.

### Data Extraction, Intercoder Reliability, and Quality Assessment

The required information for all included technologies and studies was coded by the first author using a data extraction form. Owing to the large variety of study designs, the included studies were assessed for quality (detailed scores given in Multimedia Appendix 1) using the tool by Hawker et al [108]. Using this tool, the *abstract and title*, *introduction and aims*, *method and data*, *sample size* (if applicable), *data analysis*, *ethics and bias*, *results*, *transferability or generalizability*, and *implications and usefulness* were allocated a score between 1 and 4, with higher scoring studies indicating higher quality. A random selection with 14% (10/73) of the papers was listed in a spreadsheet and coded by a second researcher following the defined coding categories and subcategories. The decisions made by the second researcher were compared with the first. With the recommended items (Multimedia Appendix 2), there was only one small disagreement between *physical activity* and *leisure activity* [32], but all other recommended items were rated exactly the same; the recommender techniques had a Cohen κ value of 0.71 (*P*<.001) and the evaluation approach scored a Cohen κ value of 0.81 (*P*<.001). There was moderate agreement (Cohen κ=0.568; *P*<.001) between the researchers concerning the quality of the papers. The interfaces used were in perfect agreement. Finally, the coding data are available in Multimedia Appendix 1.

## Results

### Study Details

**Figure 1:**
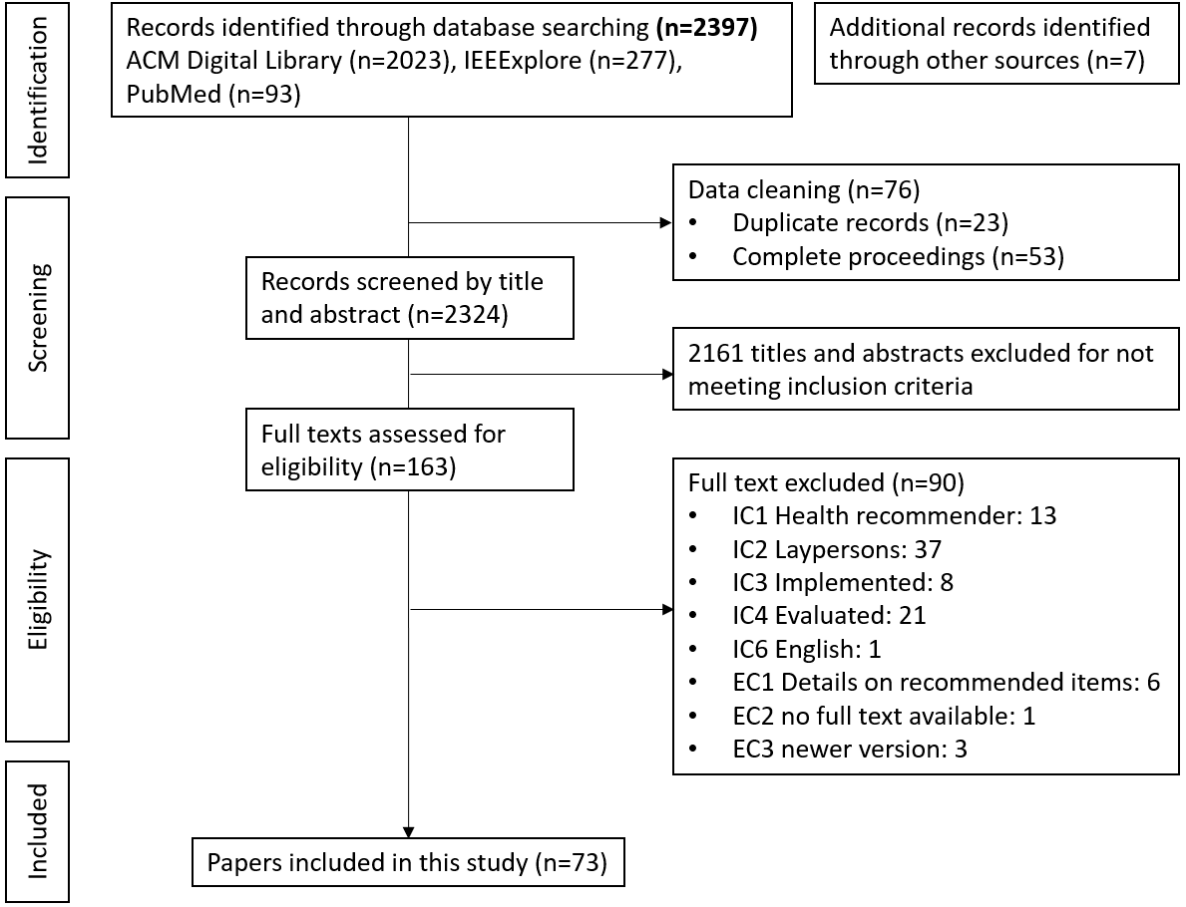
Flow diagram according to the PRISMA (Preferred Reporting Items for Systematic Reviews and Meta-Analyses) guidelines. EC: exclusion criteria; IC: inclusion criteria.

The literature in three databases yielded 2340 studies, of which only 23 were duplicates and 53 were full proceedings, leaving 2324 studies to be screened for eligibility. A total of 2161 studies were excluded upon title or abstract screening because they were unrelated to health or targeted at medical professionals or because the papers did not report an evaluation. Thus, the remaining 163 full-text studies were assessed for eligibility. After the removal of 90 studies that failed the inclusion criteria or met the exclusion criteria, 73 published studies remained. The search process is illustrated in Figure 1.

**Figure 2:**
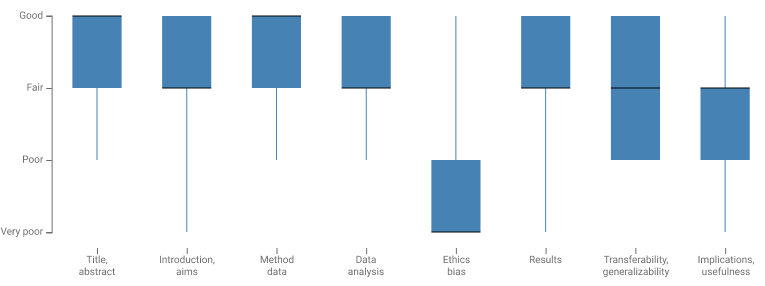
Distribution of the quality assessment using Hawker tool.

All included papers were published in 2009 or later, following an upward trend of increased popularity. The publication venues of HRSs are diverse. Only the PervasiveHealth [33-35], RecSys [36,37,109], and WI-IAT [38-40] conferences published 3 papers each that were included in this study. The *Journal of Medical Internet Research* was the only journal that occurred more frequently in our data set; 5 papers were published by *Journal of Medical Internet Research* [41-45]. The papers were first rated using Hawker tool [108]. Owing to a large number of offline evaluations, we did not include the sample score to enable a comparison between all included studies. The papers received an average score of 24.32 (SD 4.55, max 32; data set presented in Multimedia Appendix 1). Most studies scored *very poor* on reporting ethics and potential biases, as illustrated in Figure 2. However, there is an upward trend over the years in more adequate reporting of ethical issues and potential biases. The authors also limited themselves to their specific case studies and did not make any recommendations for policy (last box plot is presented in Figure 2). All 73 studies reported the use of different data sets. Although all recommended items were health related, only Asthana et al [46] explicitly mentioned using electronic health record data. Only 14% (10/73) [7,47-55] explicitly reported that they addressed the cold-start problem.

### Recommended Items

#### Overview

Most HRSs operated in different domains and thus recommended different items. In this study, four nonmutually exclusive categories of recommended items were identified: lifestyle 33% (24/73), nutrition 36% (26/73), general health information 32% (23/73), and specific health condition–related recommendations 12% (9/73). The only significant trend we found is the increasing popularity of *nutrition advice*. Multimedia Appendix 2 shows the distribution of these recommended items.

#### Lifestyle

Many HRSs, 33% (24/73) of the included studies, suggest lifestyle-related items, but they differ greatly in their exact recommendations. Physical activity is often recommended. Physical activities are often personalized according to personal interests [56] or the context of the user [35]. In addition to physical activities, Kumar et al [32] recommend eating, shopping, and socializing activities. One study analyzes the data and measurements to be tracked for an individual and then recommends the appropriate wearable technologies to stimulate proactive health [46]. A total of 7 studies [7,9,42,53,57-59] more directly try to convince users to alter their behavior by recommending them to change, or alter their behavior: for example, Rabbi et al [7] learn “a user’s physical activity and dietary behavior and strategically suggests changes to those behaviors for a healthier lifestyle*.*” In another example, both Marlin et al [59] and Sadasivam et al [42] motivate users to stop smoking by providing them with tailored messages, such as “Keep in mind that cravings are temporary and will pass.” Messages could reflect the theoretical determinants of quitting, such as positive outcome expectations and self-efficacy enhancing small goals [42].

#### Nutrition

The influence of food on health is also clear from the large subset of HRSs dealing with nutrition recommendations. A mere 36% (26/73) of the studies recommend nutrition-related information, such as recipes [50], meal plans [36], restaurants [60], or even help with choosing healthy items from a restaurant menu [61]. Wayman and Madhvanath [37] provide automated, personalized, and goal-driven dietary guidance to users based on grocery receipt data. Trattner and Elsweiler [62] use postfiltering to focus on healthy recipes only and extended them with nutrition advice, whereas Ge et al [48] require users to first enter their preferences for better recommendations. Moreover, Gutiérrez et al [63] propose healthier alternatives through augmented reality when the users are shopping. A total of 7 studies specifically recommend healthy recipes [47,48,50,62,64-66]. Most HRSs consider the health condition of the user, such as the DIETOS system [67]. Other systems recommend recipes that are synthesized based on existing recipes and recommend new recipes [64], assist parents in making appropriate food for their toddlers [47], or help users to choose allergy-safe recipes [65].

#### General Health Information

According to 32% (23/73) of the included studies, providing access to trustworthy health care information is another common objective. A total of 5 studies focused on personalized, trustworthy information per se [15,55,68-70], whereas 5 others focused on guiding users through health care forums [52,71-74]. In total, 3 studies [55,68,69] provided personalized access to general health information. For example, Sanchez Bocanegra et al [15] targeted health-related videos and augmented them with trustworthy information from the United States National Library of Medicine (MedlinePlus) [110]. A total of 3 studies [52,72,74] related to health care forums focused on finding relevant threads. Cho et al [72] built “an autonomous agent that automatically responds to an unresolved user query by posting an automated response containing links to threads discussing similar medical problems.” However, 2 studies [71,73] helped patients to find similar patients. Jiang and Yang [71] investigated approaches for measuring user similarity in web-based health social websites, and Lima-Medina et al [73] built a virtual environment that facilitates contact among patients with cardiovascular problems. Both studies aim to help users seek informational and emotional support in a more efficient way. A total of 4 studies [41,75-77] helped patients to find appropriate doctors for a specific health problem, and 4 other studies [51,78-80] focused on finding nearby hospitals. A total of 2 studies [78,79] simply focused on the clinical preferences of the patients, whereas Krishnan et al [111] “provide health care recommendations that include Blood Donor recommendations and Hospital Specialization.” Finally, Tabrizi et al [80] considered patient satisfaction as the primary feature of recommending hospitals to the user.

#### Specific Health Conditions

The last group of studies (9/73, 12%) focused on specific health conditions. However, the recommended items vary significantly. Torrent-Fontbona and Lopez Ibanez [81] have built a knowledge-based recommender system to assist diabetes patients in numerous cases, such as the estimated carbohydrate intake and past and future physical activity. Pustozerov et al [43] try to “reduce the carbohydrate content of the desired meal by reducing the amount of carbohydrate-rich products or by suggesting variants of products for replacement.” Li and Kong [82] provided diabetes-related information, such as the need for a low-sodium lunch, targeted on American Indians through a mobile app. Other health conditions supported by recommender systems include depression and anxiety [83], mental disorders [45], and stress [34,54,84,85]. Both the mental disorder [45] and the depression and anxiety [83] HRSs recommend mobile apps. For example, the app MoveMe suggests exercises tailored to the user’s mood. The HRS to alleviate stress includes recommending books to read [54] and meditative audios [85].

### Recommender Techniques

#### Overview

The recommender techniques used varied greatly. Table 2 shows the distributions of these recommender techniques.

**Table 2:** Overview of the different recommender techniques used in the studies.

Main technique^a^	Study	Total studies, n (%)
Collaborative filtering	[59,69,76]	3 (4)
Content-based filtering	[15,32,54,63,72,86,87]	7 (10)
Knowledge-based filtering	[9,38,44,50,57,64,66,68,79,81,82,84,88-91]	16 (22)
Hybrid	[7,29,34,36,37,39-41,43,46-48,53,55,56,61,65,67,69,70,73,74,77,78,80,85,92-96,111]	32 (44)
Context-based techniques	[33,35,58,97]	4 (5)
Not specified	[45,83,98]	3 (4)
Comparison between techniques	[8,49,52,60,62,71,75,99]	8 (11)

^a^The papers are classified based on how the authors reported their techniques.

#### Recommender Techniques in Practice

The majority of HRSs (49/73, 67%) rely on knowledge-based techniques, either directly (17/49, 35%) or in a hybrid approach (32/49, 65%). Knowledge-based techniques are often used to incorporate additional information of patients into the recommendation process [112] and have been shown to improve the quality of recommendations while alleviating other drawbacks such as cold-start and sparsity issues [14]. Some studies use straightforward approaches, such as if-else reasoning based on domain knowledge [9,79,81,82,88,90,100]. Other studies use more complex algorithms such as particle swarm optimization [57], fuzzy logic [68], or reinforcement algorithms [44,84].

In total, 32 studies reported using a combination of recommender techniques and are classified as *hybrid recommender systems*. Different knowledge-based techniques are often combined. For example, Ali et al [56] used a combination of rule-based reasoning, case-based reasoning, and preference-based reasoning to recommend personalized physical activities according to the user’s specific needs and personal interests. Asthana et al [46] combined the knowledge of a decision tree and demographic information to identify the health conditions. When health conditions are known, the system knows which measurements need to be monitored. A total of 7 studies used a *content-based technique* to recommend educational content [15,72,87], activities [32,86], reading materials [54], or nutritional advice [63].

Although collaborative filtering is a popular technique [113], it is not used frequently in the HRS domain. Marlin et al [59] used collaborative filtering to personalize future smoking cessation messages based on explicit feedback on past messages. This approach is used more often in combination with other techniques. A total of 2 studies [38,92] combined content-based techniques with collaborative filtering. Esteban et al [92], for instance, switched between content-based and collaborative approaches. The former approach is used for new physiotherapy exercises and the latter, when a new patient is registered or when previous recommendations to a patient are updated.

#### Context-Based Recommender Techniques

From an HRS perspective, context is described as an aggregate of various information that describes the setting in which an HRS is deployed, such as the location, the current activity, and the available time of the user. A total of 5 studies use contextual information to improve their recommendations but use a different technique; a prefilter uses contextual information to select or construct the most relevant data for generating recommendations. For example, in Narducci et al [75], the set of potentially similar patients was restricted to consultation requests in a specific medical area. Rist et al [33] applied a rule-based contextual prefiltering approach [114] to filter out inadequate recommendations, for example, “if it is dark outside, all outdoor activities, such as ‘take a walk,’ are filtered out” [33] before they are fed to the recommendation algorithm. However, a postfilter removes the recommended items *after* they are generated, such as filtering outdoor activities while it is raining. Casino et al [97] used a postfiltering technique by running the recommended items through a *real-time constraint checker*. Finally, contextual modeling, which was used by 2 studies [35,58], uses contextual information directly in the recommendation function as an explicit predictor of a user’s rating for an item [114].

Location, agenda, and weather are examples of contextual information used by Lin et al [35] to promote the adoption of a healthy and active lifestyle. Cerón-Rios et al [58] used a decision tree to analyze user needs, health information, interests, time, location, and lifestyle to promote healthy habits. Casino et al [97] gathered contextual information through smart city sensor data to recommend healthier routes. Similarly, contextual information was acquired by Rist et al [33] using sensors embedded in the user’s environment.

#### Comparisons

A total of 8 papers compared different recommender techniques to find the most optimal algorithm for a specific data set, end users, domain, and goal. Halder et al [52] used two well-known health forum data sets (PatientsLikeMe [115] and HealthBoards [116]) to compare 7 recommender techniques (among collaborative filtering and content-based filtering) and found that a hybrid approach scored best [52]. Another example is the study by Narducci et al [75], who compared four recommendation algorithms: cosine similarity as a baseline, collaborative filtering, their own *HealthNet* algorithm, and a hybrid of HealthNet and cosine similarity. They concluded that a prefiltering technique for similar patients in a specific medical area can drastically improve the recommendation accuracy [75]. The average and SD of the resulting ratings of the two collaborative techniques are compared with random recommendations by Li et al [60]. They show that a hybrid approach of a collaborative filter augmented with the calculated health level of the user performs better. In their nutrition-based meal recommender system, Yang et al [49] used item-wise and pairwise image comparisons in a two-step process. In conclusion, the 8 studies showed that recommendations can be improved when the benefits of multiple recommender techniques are combined in a hybrid solution [60] or contextual filters are applied [75].

### Evaluation Approach

#### Overview

HRSs can be evaluated in multiple ways. In this study, we found two categories of HRS evaluations: (1) offline evaluations that use computational approaches to evaluate the HRS and (2) evaluations in which an end user is involved. Some studies used both, as shown in Multimedia Appendix 3.

#### Offline Evaluations

Of the total studies, 47% (34/73) do not involve users directly in their method of evaluation. The evaluation metrics also vary greatly, as many distinct metrics are reported in the included papers (Multimedia Appendix 3). Precision 53% (18/34), accuracy 38% (13/34), performance 35% (12/34), and recall 32% (11/34) were the most commonly used offline evaluation metrics. Recall has been used significantly more in recent papers, whereas accuracy also follows an upward trend. Moreover, performance was defined differently across studies. Torrent-Fontbona and Lopez Ibanez [81] compared the “amount of time in the glycaemic target range by reducing the time below the target” as performance. Cho et al [72] compared the precision and recall to report the performance. Clarke et al [84] calculated their own reward function to compare different approaches, and Lin et al [35] measured *system performance* as the number of messages sent in their in the wild study. Finally, Marlin et al [59] tested the predictive performance using a triple cross-validation procedure.

Other popular offline evaluation metrics are accuracy-related measurements, such as mean absolute (percentage) error, 18% (6/34); normalized discounted cumulative gain (nDCG), 18% (6/34); *F*_1_ score, 15% (5/34); and root mean square error, 15% (5/34). The other metrics were measured inconsistently. For example, Casino et al [97] reported that they measure robustness but do not outline what they measure as robustness. However, they measured the mean absolute error. Torrent-Fontbona and Lopez Ibanez [81] defined robustness as the capability of the system to handle missing values. Effectiveness is also measured with different parameters, such as its ability to take the right classification decisions [75] or in terms of key opinion leaders’ identification [41]. Finally, Li and Zaman [68] measured trust with a proxy: “evaluate the trustworthiness of a particular user in a health care social network based on factors such as role and reputation of the user in the social community” [68].

#### User Evaluations

##### Overview

Of the total papers, 53% (39/73) included participants in their HRS evaluation, with an average sample size of 59 (SD 84) participants (excluding the outlier of 8057 participants, as recruited in the study by Cheung et al [83]). On average, studies ran for more than 2 months (68, SD 56 days) and included all age ranges. There is a trend of increasing sample size and study duration over the years. However, only 17 studies reported the study duration; therefore, these trends were not significant. Surveys (12/39, 31%), user studies (10/39, 26%), and deployments in the wild (10/39, 26%) were the most used user evaluations. Only 6 studies used an RCT to evaluate their HRS. Finally, although all the included studies focused on HRSs and were dealing with sensitive data, only 12% (9/73) [9,34,42-45,73,83,95] reported ethical approval by a review board.

##### Surveys

No universal survey was found, as all the studies deployed a distinct survey. Ge et al [48] used the system usability scale and the framework of Knijnenburg et al [117] to explain the user experience of recommender systems. Esteban et al [95] designed their own survey with 10 questions to inquire about user experience. Cerón-Rios [58] relied on the ISO/IEC (International Organization of Standardization/International Electrotechnical Commission) 25000 standard to select 7 usability metrics to evaluate usability. Although most studies did not explicitly report the surveys used, user experience was a popular evaluation metric, as in the study by Wang et al [69]. Other metrics range from measuring user satisfaction [69,99] and perceived prediction accuracy [59] (with 4 self-composed questions). Nurbakova et al [98] combined data analytics with surveys to map their participants’ psychological background, including orientations to happiness measured using the Peterson scale [118], personality traits using the Mini-International Personality Item Pool [119], and Fear of Missing Out based on the Przybylski scale [120].

##### Single-Session Evaluations (User Studies)

A total of 10 studies recruited users and asked them to perform certain tasks in a single session. Yang et al [49] performed a 60-person user study to assess its feasibility and effectiveness. Each participant was asked to rate meal recommendations relative to those made using a traditional survey-based approach. In a study by Gutiérrez et al [63], 15 users were asked to use the health augmented reality assistant and measure the qualities of the recommender system, users’ behavioral intentions, perceived usefulness, and perceived ease of use. Jiang and Xu [77] performed 30 consultations and invited 10 evaluators majoring in medicine and information systems to obtain an average rating score and nDCG. Radha et al [8] used comparative questions to evaluate the feasibility. Moreover, Cheng et al [89] used 2 user studies to rank two *degrees of compromise* (DOC). A low DOC assigns more weight to the algorithm, and a high DOC assigns more weight to the user’s health perspective. Recommendations with a lower DOC are more efficient for the user’s health, but recommendations with a high DOC could convince users to believe that the recommended action is worth doing. Other approaches used are structured interviews [58], ranking [86,89], asking for unstructured feedback [40,88], and focus group discussions [87]. Finally, 3 studies [15,75,90] evaluated their system through a heuristic evaluation with expert users.

##### In the Wild

Only 2 studies tested their HRS *into the wild* recruited patients (people with a diagnosed health condition) in their evaluation. Yom-Tov et al [44] provided 27 sedentary patients with type 2 diabetes with a smartphone-based pedometer and a personal plan for physical activity. They assessed the effectiveness by calculating the amount of activity that the patient performed after the last message was sent. Lima-Medina et al [73] interviewed 45 patients with cardiovascular problems after a 6-month study period to measure (1) social management results, (2) health care plan results, and (3) recommendation results. Rist et al [33] performed an in-situ evaluation in an apartment of an older couple and used the data logs to describe the usage but augmented the data with a structured interview.

Yang et al [49] conducted a field study of 227 anonymous users that consisted of a training phase and a testing phase to assess the prediction accuracy. Buhl et al [99] created three user groups according to the recommender technique used and analyzed log data to compare the response rate, open email rate, and consecutive log-in rate. Similarly, Huang et al [76] compared the ratio of recommended doctors chosen and reserved by patients with the recommended doctors. Lin et al [35] asked 6 participants to use their HRSs for 5 weeks, measured system performance, studied user feedback to the recommendations, and concluded with an open-user interview. Finally, Ali et al [56] asked 10 volunteers to use their weight management systems for a couple of weeks. However, they do not focus on user-centric evaluation, as “only a prototype of the [...] platform is implemented.”

Rabbi et al [7] followed a single case with multiple baseline designs [121]. Single-case experiments achieve sufficient statistical power with a large number of repeated samples from a single individual. Moreover, Rabbi et al [7] argued that HRSs suit this requirement “since enough repeated samples can be collected with automated sensing or daily manual logging [121].” Participants were exposed to 2, 3, or 4 weeks of the control condition. The study ran for 7-9 weeks to compensate for the novelty effects. Food and exercise log data were used to measure changes in food calorie intake and calorie loss during exercise.

##### Randomized Controlled Trials

Only 6 studies followed an RCT approach. In the RCT by Bidargaddi et al [45], a large group of patients (n=192) and control group (n=195) were asked to use a web-based recommendation service for 4 weeks that recommended mental health and well-being mobile apps. Changes in well-being were measured using the Mental Health Continuum-Short Form [122]. The RCT by Sadasivam et al [42] enrolled 120 current smokers (n=74) and control group (n=46) as a follow-up to a previous RCT [123] that evaluated their portal to specifically evaluate the HRS algorithm. Message ratings were compared between the intervention and control groups.

Cheung et al [83] measured app loyalty through the number of weekly app sessions over a period of 16 weeks with 8057 users. In the study by Paredes et al [34], 120 participants had to use the HRS for at least 26 days. Self-reported stress assessment was performed before and after the intervention. Agapito et al [67] used an RCT with 40 participants to validate the sensitivity (true positive rate/[true positive rate+false negative rate]) and specificity (true negative rate/[true negative rate+false positive rate]) of the DIETOS HRS. Finally, Luo et al [93] performed a small clinical trial for more than 3 months (but did not report the number of participants). Their primary outcome measures included two standard clinical blood tests: fasting blood glucose and laboratory-measured glycated hemoglobin, before and after the intervention.

#### Interface

##### Overview

**Figure 3:**
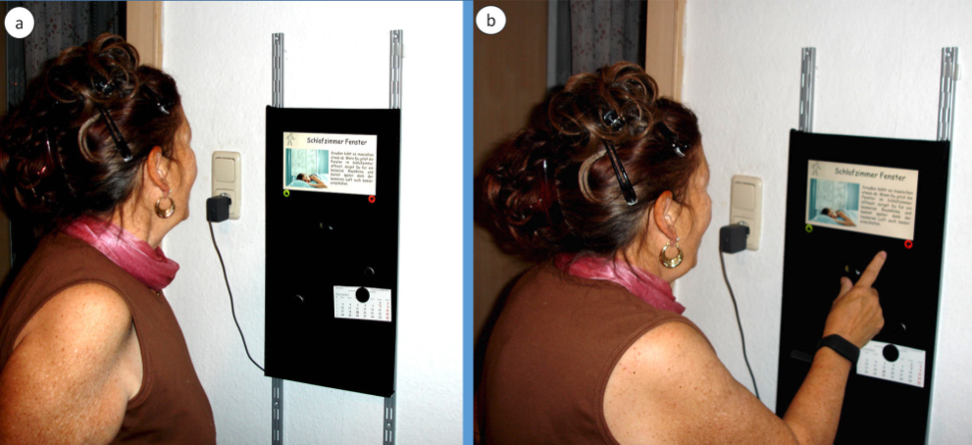
Rist et al installed a kiosk in the home of older adults as a direct interface to their health recommender system.

**Figure 4:**
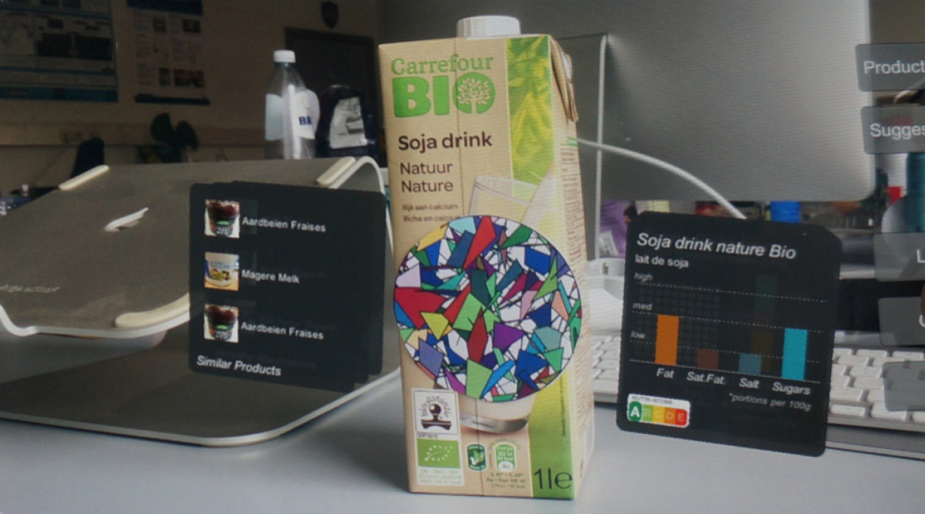
An example of the recommended healthy alternatives by Gutiérrez et al.

Only 47% (34/73) of the studies reported implementing a graphical user interface to communicate the recommended health items to the user. As illustrated in Table 3, 53% (18/34) use a mobile interface, usually through a mobile (web) app, whereas 36% (14/34) use a web interface to show the recommended items. Rist et al [33] built a kiosk into older adults’ homes, as illustrated in Figure 3. Gutiérrez et al [63] used Microsoft HoloLens to project healthy food alternatives in augmented reality surrounding a physical object that the user holds, as shown in Figure 4.

**Table 3:** Distribution of the interfaces used among the different health recommender systems (n=34).

Interface	Study	Total studies, n (%)
Mobile	[7,34,35,40,44,48,56,58,66,69,77,78,82-84,86,88,97]	18 (53)
Web	[9,15,37,41,45,49,61,70,73,75,79,85,90,95]	14 (41)
Kiosk	[33]	1 (3)
HoloLens	[63]	1 (3)

##### Visualization

A total of 7 studies [33,34,37,63,79,88,97] or approximately one-fourth of the studies with an interface included visualizations. However, the approach used was different for all studies, as shown in Table 4. Showing stars to show the relevance of a recommended item are only used by Casino et al [97] and Gutiérrez et al [63]. Wayman and Madhvanath [37] also used bar charts to visualize the progress toward a health goal. They visualize the healthy proportions, that is, what the user should eat. Somewhat more complex visualizations are used by Ho and Chen [88] who visualized the user’s ECG zones. Paredes et al [34] presented an emotion graph as an input screen. Rist et al [33] visualized an example of how to perform the recommended activity.

**Table 4:** Distribution of the visualizations used among the different health recommender systems (n=7).

Visualization technique	Study	Total studies, n (%)
Bar charts	Wayman and Madhvanath [37] and Gutiérrez et al [63]	2 (29)
Heatmap	Ho and Chen [88]	1 (14)
Emotion graph	Paredes et al [34]	1 (14)
Visual example of action	Rist et al [33]	1 (14)
Map	Avila-Vazquez et al [79]	1 (14)
Star rating	Casino et al [97]	1 (14)

##### Transparency

In the study by Lage et al [87], participants expressed that:

they would like to have more control over recommendations received. In that sense, they suggested more information regarding the reasons why the recommendations are generated and more options to assess them.

A total of 7 studies [7,37,41,45,63,66,82] explained the reasoning behind recommendations to end users at the user interface. Gutiérrez et al [63] provided recommendations for healthier food products and mentioned that the items (Figure 4) are based on the users’ profile. Ueta et al [66] explained the relationship between the recommended dishes and a person’s health conditions. For example, a person with acne can see the following text: “15 dishes that contained Pantothenic acid thought to be effective in acne a lot became a hit” [66]. Li and Kong [82] showed personalized recommended health actions in a message center. Color codes are used to differentiate between reminders, missed warnings, and recommendations. Rabbi et al [7] showed tailored motivational messages to explain why activities are recommended. For example, when the activity *walk near East Ave* is recommended, the app shows the additional message:

1082 walks in 240 days, 20 mins of walk everyday. Each walk nearly 4 min. Let us get 20 mins or more walk here today

7

Wayman and Madhvanath [37] first visualized the user’s personal nutrition profile and used the lower part of the interface to explain why the item was recommended. They provided an illustrative example of spaghetti squash. The explanation shows that:

This product is high in Dietary_fiber, which you could consume more of. Try to get 3 servings a week

37

**Figure 5:**
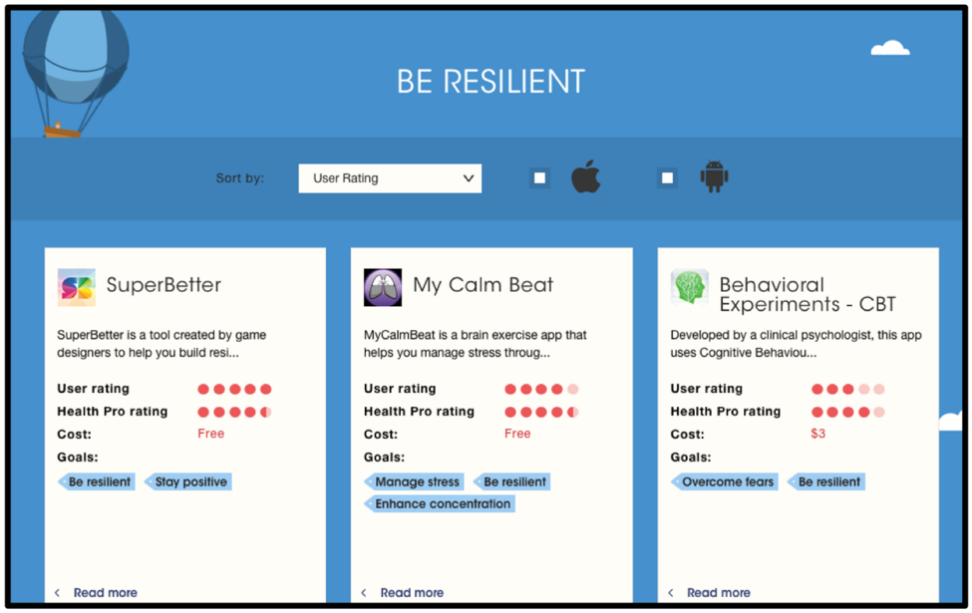
A screenshot from the health recommender system of Bidargaddi et al. Note the blue tags illustrating how each recommended app matches the users’ goals.

Guo et al [41] recommended doctors and showed a horizontal bar chart to visualize the user’s values compared with the average values. Finally, Bidargaddi et al [45] visualized how the recommended app overlaps with the goal set by the users, as illustrated in Figure 5.

## Discussion

### Principal Findings

HRSs cover a multitude of subdomains, recommended items, implementation techniques, evaluation designs, and means of communicating the recommended items to the target user. In this systematic review, we clustered the recommended items into four groups: lifestyle, nutrition, general health care information, and specific health conditions. There is a clear trend toward HRSs that provide *well-being* recommendations but do not directly intervene in the user’s medical status. For example, almost 70% (50/73; lifestyle and nutrition) focused on no strict medical recommendations. In the lifestyle group, physical activities (10/24, 42%) and advice on how to potentially change behavior (7/24, 29%) were recommended most often. In the nutrition group, these recommendations focused on nutritional advice (8/26, 31%), diets (7/26, 27%), and recipes (7/26, 27%). A similar trend was observed in the health care information group, where HRSs focused on guiding users to the appropriate environments such as hospitals (5/23, 22%) and medical professionals (4/23, 17%) or on helping users find qualitative information (5/23, 22%) on validated sources or from experiences by similar users and patients on health care forums (3/23, 13%). Thus, they only provide general information and do not intervene by recommending, for example, changing medication. Finally, when HRSs targeted specific health conditions, they recommended nonintervening actions, such as meditation sessions [84] or books to read [54].

Although collaborative filtering is commonly the most used technique in other domains [124], here only 3 included studies reported the use of a collaborative filtering approach. Moreover, 43% (32/73) of the studies applied a hybrid approach, showing that HRS data sets might need special attention, which might also be the reason why all 73 studies used distinct data sets. In addition, the HRS evaluations varied greatly and were divided over evaluations where the end user was involved and evaluations that did not evolve users (offline evaluations). Only 47% (34/73) of the studies reported implementing a user interface to communicate recommendations to the user, despite the need to show the rationale of recommendations, as echoed by many researchers and practitioners [11]. Moreover, only 15% (7/47) included a (basic) visualization.

Unfortunately, this general lack of agreement on how to report HRSs might introduce researcher bias, as a researcher is currently completely unconstrained in defining what and how to measure the added value of an HRS. Therefore, further debate in the health recommender community is needed on how to define and measure the impact of HRSs. On the basis of our review and contribution to this discussion, we put forward a set of essential information that researchers should report in their studies.

### Considerations for Practice

**Figure 6:**
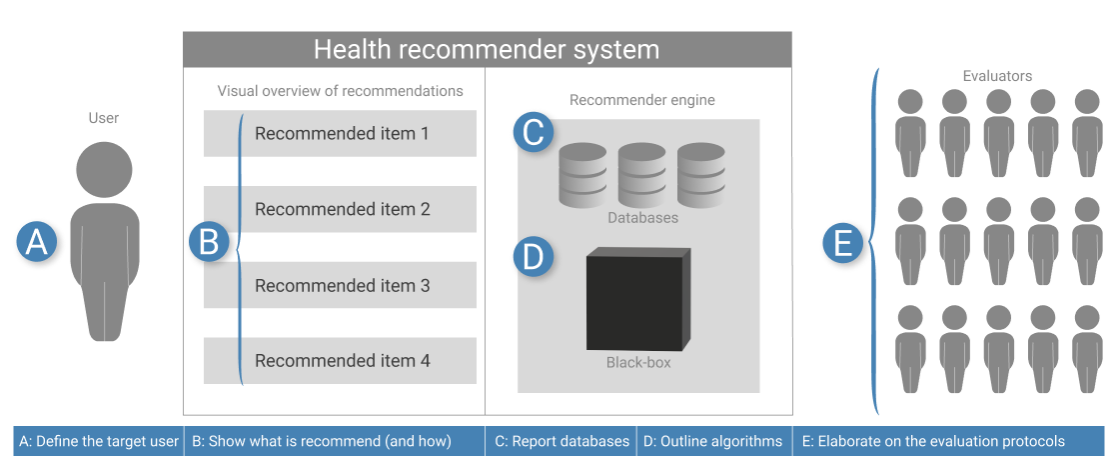
A reference frame to report health recommender system studies. On the basis of the results of this study, we suggest that it should be clear what and how items are recommended (A), who the target user is (B), which data are used (C), and which recommender techniques are applied (D). Finally, the evaluation design should be reported in detail (E).

The previously discussed results have direct implications in practice and provide suggestions for future research. Figure 6 shows a reference frame of these requirements that can be used in future studies as a quality assessment tool.

#### Define the Target User

As shown in this review, HRSs are used in a plethora of subdomains and each domain has its own experts. For example, in nutrition, the expert is most likely a dietician. However, the user of an HRS is usually a layperson without the knowledge of these domain experts, who often have different viewing preferences [125]. Furthermore, each user is unique. All individuals have idiosyncratic reasons for why they act, think, behave, and feel in a certain way at a specific stage of their life [126]. Not everybody is motivated by the same elements. Therefore, it is important to know the target user of the HRS. What is their previous knowledge, what are their goals, and what motivates them to act on a recommended item?

#### Show What Is Recommended (and How)

Researchers have become aware that *accuracy* is not sufficient to increase the effectiveness of a recommender system [127]. In recent years, research on human factors has gained attention. For example, He et al [11] surveyed 24 existing interactive recommender systems and compared their transparency, justification, controllability, and diversity. However, none of these 24 papers discussed HRSs. This indicates the gap between HRSs and recommender systems in other fields. Human factors have gained interest in the recommender community by “combining interactive visualization techniques with recommendation techniques to support transparency and controllability of the recommendation process” [11]. However, in this study, only 10% (7/73) explained the rationale of recommendations and only 10% (7/73) included a visualization to communicate the recommendations to the user. We do not argue that all HRSs should include a visualization or an explanation. However, researchers should pay attention to the delivery of these recommendations. Users need to understand, believe, and trust the recommended items before they can act on it.

To compare and assess HRSs, researchers should unambiguously report what the HRS is recommending. After all, typical recommender systems act like a *black box*, that is, they show suggestions without explaining the provenance of these recommendations [11]. Although this approach is suitable for typical e-commerce applications that involve little risk, transparency is a core requirement in higher risk application domains such as health [128]. Users need to understand why a recommendation is made, to assess its value and importance [12]. Moreover, health information can be cumbersome and not always easy to understand or situate within a specific health condition [129]. Users need to know whether the recommended item or action is based on a trusted source, tailored to their needs, and actionable [130].

#### Report the Data Set Used

All 73 studies used a distinct data set. Furthermore, some studies combine data from multiple databases, making it even more difficult to judge the quality of the data [35]. Nonetheless, most studies use self-generated data sets. This makes it difficult to compare and externally validate HRSs. Therefore, we argued that researchers should clarify the data used and potentially share whether these data are publicly available. However, in health data are often highly privacy sensitive and cannot be shared among researchers.

#### Outline the Recommender Techniques

The results show that there is no panacea for which recommender technique to use. The included studies differ from logic filters to traditional recommender techniques, such as collaborative filtering and content-based filtering to hybrid solutions and self-developed algorithms. However, with 44% (32/73), there is a strong trend toward the use of hybrid recommender techniques. The low number of collaborative filter techniques might be related to the fact that the evaluation sample sizes were also relatively low. Unfortunately, some studies have not fully disclosed the techniques used and only reported on the main algorithm used. It is remarkable that studies published in high-impact journals, such as studies by Bidargaddi et al [45] and Cheung et al [83], did not provide information on the recommender technique used. Nonetheless, disclosing the recommender technique allows other researchers not only to build on empirically tested technologies but also to verify whether key variables are included [29]. User data and behavior data can be identified to augment theory-based studies [29]. Researchers should prove that the algorithm is capable of recommending valid and trustworthy recommendations to the user based on their available data set.

#### Elaborate on the Evaluation Protocols

HRSs can be evaluated using different evaluation protocols. However, the protocol should be outlined mainly by the research goals of the authors. On the basis of the papers included in this study, we differentiate between the two approaches. In the first approach, the authors aim to influence their users’ health, for example, by providing personalized diabetes guidelines [81] or prevention exercises for users with low back pain [95]. Therefore, the end user should always be involved in both the design and evaluation processes. However, only 8% (6/73) performed an RCT and 14% (10/73) deployed their HRS in the wild. This lack of user involvement has been noted previously by researchers and has been identified as a major challenge in the field [27,28]. Nonetheless, in other domains, such as job recommenders [131] or agriculture [132], user-centered design has been proposed as an important methodology in the design and development of tools used by end users, with the purpose of gaining trust and promoting technology acceptance, thereby increasing adoption with end users. Therefore, we recommend that researchers evaluate their HRSs with actual users. A potential model for a user-centric approach to recommender system evaluation is the user-centric framework proposed by Knijnenburg et al [117].

Research protocols need to be elaborated and approved by an ethical review board to prevent any impact on users. Authors should report how they informed their users and how they safeguarded the privacy of the users. This is in line with the modern journal and conference guidelines. For example, editorial policies of the *Journal of Medical Internet Research* state that “when reporting experiments on human subjects, authors should indicate IRB (Institutional Rese[a]rch Board, also known as REB) approval/exemption and whether the procedures followed were in accordance with the ethical standards of the responsible committee on human experimentation” [133]. However, only 12% (9/73) reported their approval by an ethical review board. Acquiring review board approval will help the field mature and transition from small incremental studies to larger studies with representative users to make more reliable and valid findings.

In the second approach, the authors aim to design a *better* algorithm, where *better* is again defined by the authors. For example, the algorithm might perform faster, be more accurate, and be more efficient in computing power. Although the *F*_1_ score, the mean absolute error, and nDCG are well defined and known within the recommender domain, other parameters are more ambiguous. For example, the performance or effectiveness can be assessed using different measurements. However, a health parameter can be monitored, such as the duration that a user remains within healthy ranges [81]. Furthermore, it could be a predictive parameter, such as an improved precision and recall as a proxy for performance [72]. Unfortunately, this difference makes it difficult to compare health recommendation algorithms. Furthermore, this inconsistency in measurement variables makes it infeasible to report in this systematic review which recommender techniques to use. Therefore, we argue that HRS algorithms should always be evaluated for other researchers to validate the results, if needed.

### Limitations

This study has some limitations that affect its contribution. Although an extensive scope search was conducted in scientific databases and most relevant health care informatic journals, some relevant literature in other domains might have been excluded. The keywords used in the search string could have impacted the results. First, we did not include domain-specific constructs of health, such as asthma, pregnancy, and iron deficiency. Many studies may implicitly report healthy computer-generated recommendations when they research the impact of a new intervention. In these studies, however, building an HRS is often not their goal and, therefore, was excluded from this study. Second, we searched for papers that reported studying an HRS; nonincluded studies might have built an HRS but did not report it as such. Considering our RQs, we deemed it important that authors explicitly reported their work as a recommender system. To conclude, in this study, we provide a large cross-domain overview of health recommender techniques targeted to laypersons and deliver a set of recommendations that could help the field of HRS mature.

### Conclusions

This study presents a comprehensive report on the use of HRS across domains. We have discussed the different subdomains HRS applied in, the different recommender techniques used, the different manners in which they are evaluated, and finally, how they present the recommendations to the user. On the basis of this analysis, we have provided research guidelines toward a consistent reporting of HRSs. We found that although most applications are intended to improve users’ well-being, there is a significant opportunity for HRSs to inform and guide users’ health actions. Although many of the studies present a lack of a user-centered evaluation approach, some studies performed full-scale RCT evaluations or elaborated in the wild studies to validate their HRS, showing the field of HRS is slowly maturing. On the basis of this study, we argue that it should always be clear what the HRS is recommending and to whom these recommendations are for. Graphical assets should be added to show how recommendations are presented to users. Authors should also report which data sets and algorithms were used to calculate the recommendations. Finally, detailed evaluation protocols should be reported.

We conclude that the results motivate the creation of richer applications in future design and development of HRSs. The field is maturing, and interesting opportunities are being created to inform and guide health actions.

## Appendix

Multimedia Appendix 1 Coded data set of all included papers.

Multimedia Appendix 2 Overview of recommended items by 73 studies.

Multimedia Appendix 3 Overview of evaluation approaches.

## Abbreviations

DOC: degrees of compromise
HRS: health recommender system
ISO/IEC: International Organization of Standardization/International Electrotechnical Commission
nDCG: normalized discounted cumulative gain
PRISMA: Preferred Reporting Items for Systematic Reviews and Meta-Analyses
RCT: randomized controlled trial
RQ: research question
