# Supplementary figures and images for: Health Recommender Systems: Systematic Review
Source: J Med Internet Res. 2021 Jun 29;23(6):e18035. doi: 10.2196/18035 (PMC8278303; doi:10.2196/18035)

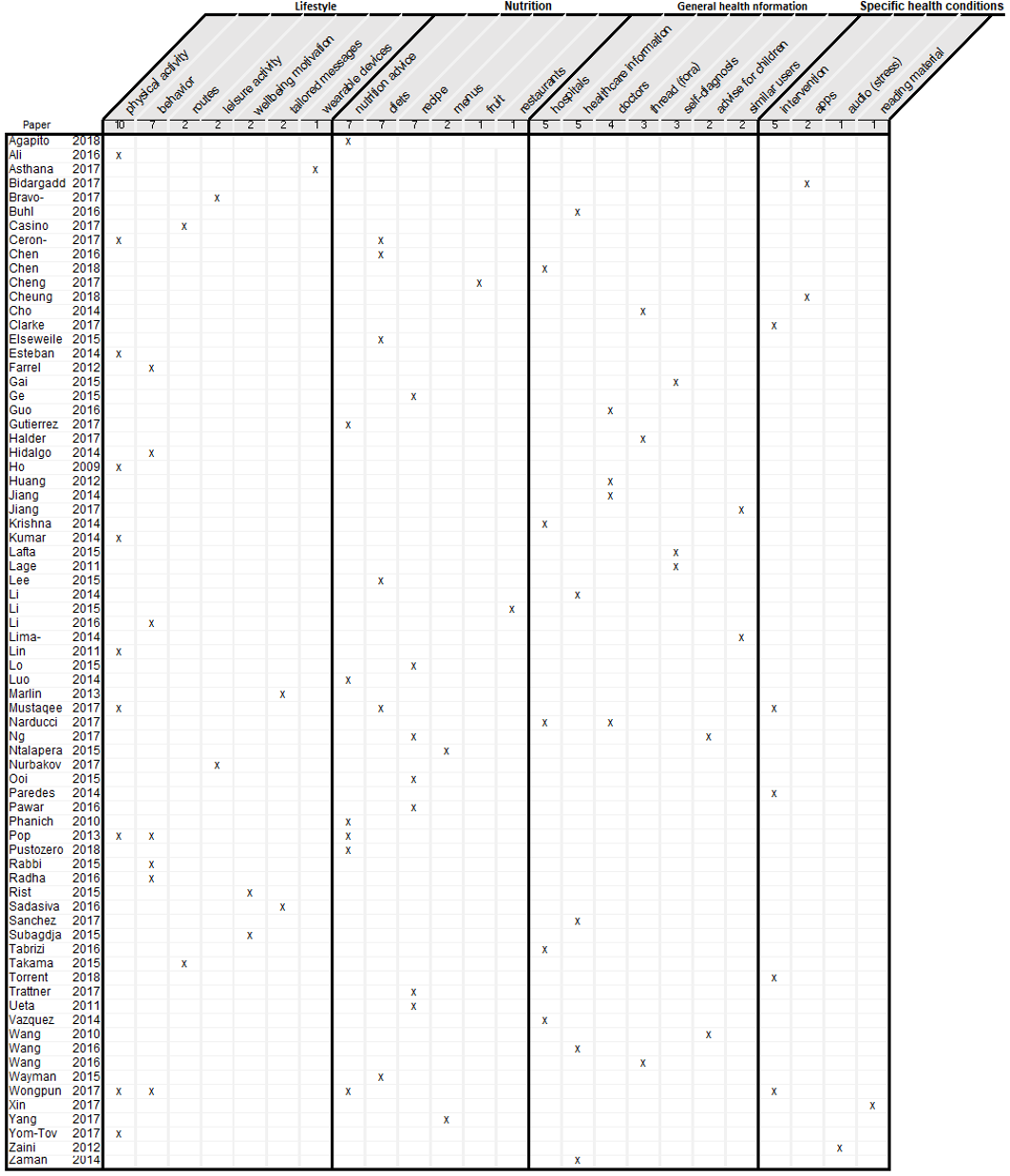

Supplement: Multimedia Appendix 2 [file jmir_v23i6e18035_app2.png]

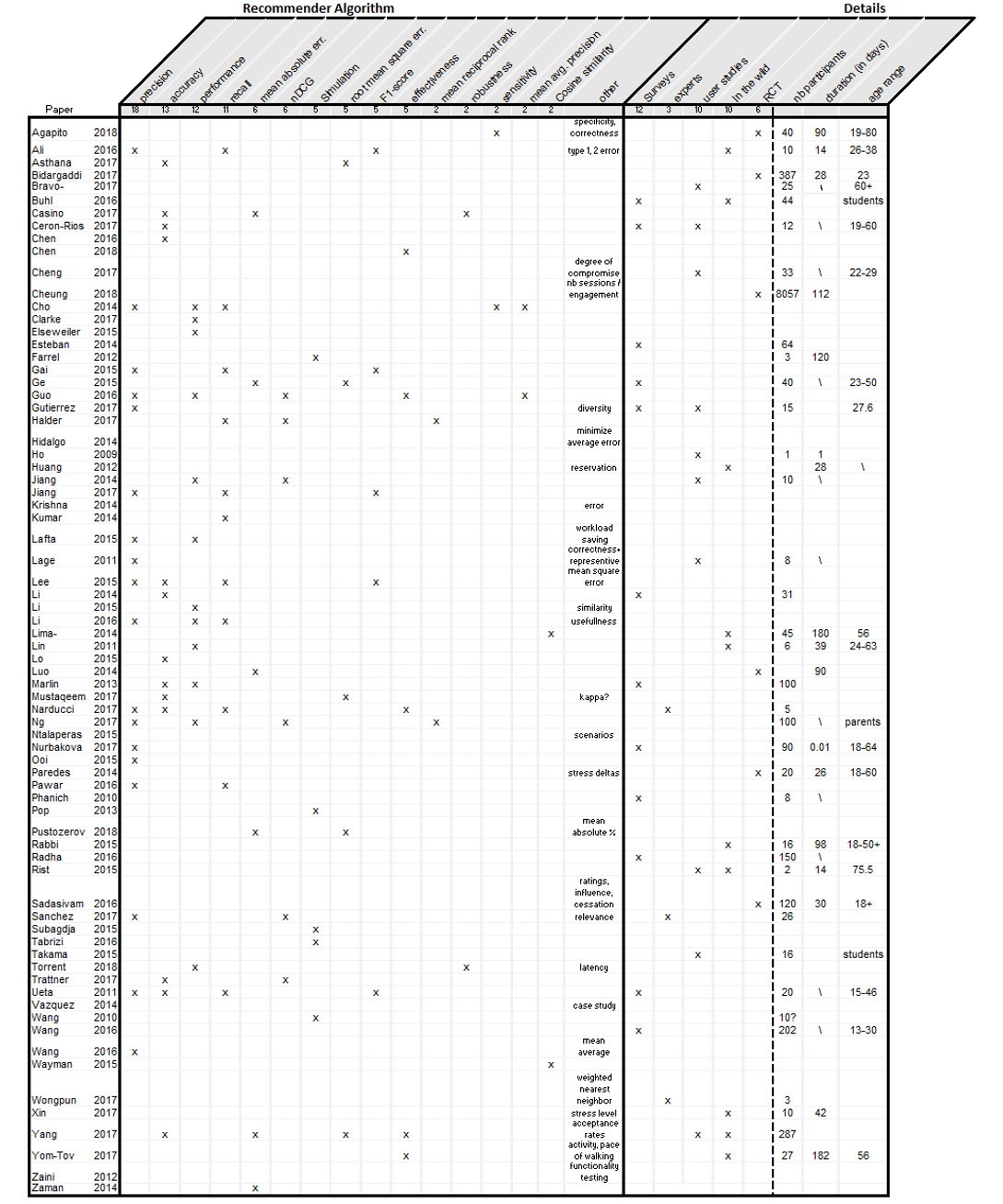

Supplement: Multimedia Appendix 3 [file jmir_v23i6e18035_app3.png]
